# Hepatitis A Virus (HAV) Recurrence and De Novo Hepatitis A Acquired at Time of Liver Transplantation: Comparison of Graft HAV Infection and Viral Kinetics

**DOI:** 10.1111/jvh.70248

**Published:** 2026-10-16

**Authors:** David Mutimer, Elinor Latham, Sowsan F. Atabani, Samreen Ijaz

**Affiliations:** ^1^ Liver and Hepatobiliary Unit, Queen Elizabeth Hospital University Hospitals Birmingham NHS Foundation Trust Birmingham UK; ^2^ Institute of Immunology and Immunotherapy College of Medical and Dental Sciences, University of Birmingham Birmingham UK; ^3^ Blood Borne Virus Unit United Kingdom Health Security Agency (UKHSA) London UK; ^4^ United Kingdom Health Security Agency (UKHSA) Specialized Microbiology and Laboratories, Southwest Bristol UK

**Keywords:** graft hepatitis, hepatitis A virus, immunosuppression, liver transplantation, transfusion‐transmitted infection, viral kinetics

## Abstract

Hepatitis A virus (HAV) infection is an uncommon indication for liver transplantation, and donor‐derived or transfusion‐associated HAV infection is rare. Data describing HAV recurrence or early acquisition following liver transplantation, particularly with longitudinal quantitative viral kinetics, are limited. We describe two liver transplant recipients with graft HAV infection occurring in different immunological contexts. One patient underwent emergency liver transplantation for HAV‐related acute liver failure and subsequently developed recurrent HAV infection of the graft. The second patient underwent liver transplantation for paracetamol‐induced acute liver failure and acquired HAV infection following peri‐operative blood transfusion in the early post‐transplant period. Serial qualitative and retrospective quantitative HAV ribonucleic acid (RNA) polymerase chain reaction (PCR) measurements were analysed alongside biochemical and clinical parameters. In the patient transplanted for fulminant HAV infection, serum HAV RNA declined rapidly after transplantation, consistent with removal of the infected native liver, followed by recurrent viraemia associated with graft hepatitis during the second and third post‐transplant months, and with viral clearance by 4 months. In contrast, the transfusion‐acquired HAV infection occurred in the setting of profound immunosuppression and absence of pre‐existing immunity, resulting in a higher peak viral load, prolonged viraemia lasting over 9 months, and more severe graft hepatitis. Viral doubling times during viraemic expansion were similar in both cases but sustained high‐level viraemia was observed only in the de novo infection. These cases provide novel insights into HAV viral kinetics following liver transplantation and highlight important differences between recurrent infection and newly acquired HAV infection in immunosuppressed recipients.

**Abbreviations**
ALT: alanine transaminase
HAV: hepatitis A virus
HBV: hepatitis B virus
HCV: hepatitis C virus
HDV: hepatitis D virus
HEV: hepatitis E virus
IgM: immunoglobulin M
NHSBT: National Health Service Blood and Transplant
PCR: polymerase chain reaction
RNA: ribonucleic acid
WHO: World Health Organization

## 1 Introduction

HAV infection is endemic in many countries, but the incidence of HAV infection in the UK is low, typically approximately 400 cases diagnosed per year [1]. Rarely, blood is donated during the presymptomatic viraemic phase of acute infection [2, 3]. The transmission event may come to light because either the blood donor or the transfusion recipient develops signs of hepatitis infection. Organ donation might also transmit HAV infection. This is also a rare event and has not been reported in the UK. Blood donors and organ donors are not screened for HAV infection, so transfusion recipients and organ‐transplanted patients may acquire HAV infection in this way.

Most HAV patients survive infection without the development of acute liver failure, and approximately 50% of patients with HAV‐related acute liver failure survive with conservative care. For patients with severe liver failure, liver transplantation may be lifesaving. Published reports of liver transplantation for this indication are few [4, 5, 6, 7], and do not include a detailed description of the recurrence of infection. However, from limited published data, it seems that chronic HAV infection of the transplant recipient does not occur or is very rare. This contrasts with the outcome of recurrent infection with hepatitis B virus (HBV) and hepatitis D virus (HDV) and hepatitis C virus (HCV) where infection of the transplanted liver without the use of antivirals for prophylaxis and treatment leads almost inevitably to aggressive recurrent chronic infection [8, 9]. Several studies have examined the viral kinetics of HBV and HCV after liver transplantation. Some focused on the kinetics of early graft reinfection and others looked at the response to antiviral treatment [10, 11, 12, 13]. Also, there is now a common experience in the diagnosis and management of hepatitis E virus (HEV) infection in immunosuppressed organ transplant recipients [14]. Even though chronic HEV infection is rarely observed in the immune competent population, the majority of immunosuppressed patients fail to eliminate HEV in the acute phase and instead progress to chronic infection with chronic liver damage.

We describe 2 liver transplant patients with hepatitis A virus infection. The first patient had acute liver failure due to HAV and underwent emergency liver transplantation which was followed by clinical and laboratory confirmed HAV recurrence. The second patient was transplanted for paracetamol poisoning and acquired HAV from transfused blood during the recovery phase after transplantation. Both patients experienced clinically significant graft hepatitis, more severe in the patient with acquired HAV than in the patient with recurrent infection. Serum HAV RNA level was measured by quantitative PCR for both patients at multiple timepoints post‐transplant.

The study was conducted in accordance with institutional ethical standards. Informed consent for publication was obtained from both patients. Quantitative HAV RNA testing was performed retrospectively on stored clinical samples without additional patient intervention. The assay was performed in the same laboratory using the same quantitative assay.

The commercially available AltoStar HAV RT‐PCR Kit 1.5 (Altona, Liverpool, UK) was used in these investigations and is provided for the qualitative detection of HAV RNA. The manufacturer states a limit of detection of 6.31 IU/mL (95% confidence interval: 4.17–11.99 IU/mL). To provide quantitative values, 10‐fold dilutions of a known HAV RNA positive control material which had been pinned against the WHO International Standard were included in each run. This provided an assay dynamic range between 3 IU/mL and 300,000 IU/mL with internal validation indicating a similar limit of detection as stated by the manufacturer. For specimens with titre greater than 300,000 IU/mL, serum dilutions were examined.

## 2 Case Reports

### 2.1 Case 1. Recurrent Hepatitis A Infection

#### 2.1.1 Clinical Picture

A 25‐year‐old Indian female came to England in June 2024. Three weeks later she was admitted to her local hospital with a brief history of vomiting, jaundice and confusion. Blood tests at presentation showed a severe acute hepatitis and the serum HAV immunoglobulin M (IgM) test was positive. The following day she was transferred to the Birmingham Queen Elizabeth Hospital. At the time of admission to the Queen Elizabeth Hospital (QEH), her laboratory tests indicated severe acute liver injury with an International Normalised Ratio (INR) of 14. The HAV IgM test was confirmed positive on the day of hospital transfer. The laboratory later confirmed that the patient was infected with HAV genotype 3A. The RNA sequence clustered with HAV sequences reported from the Indian subcontinent.

Her age and laboratory data suggested a high probability of death without transplantation (ALFA score of 1.557) [15]. She was registered immediately on the UK National Super‐urgent waiting list and underwent liver transplantation 2 days later. There was some donor‐recipient liver size disparity. Temporary external biliary drainage was established, and the recipient abdomen was closed with a silicon patch. The patient underwent a second laparotomy 2 days later when the duct‐to‐duct anastomosis was completed and permanent abdominal closure was achieved. Liver function tests were slow to settle, so liver biopsy was performed on day 19 post‐transplant. Histological changes were consistent with acute cellular rejection, and the patient received additional corticosteroid for 3 days. Liver function tests improved, and the serum alanine transaminase (ALT) and serum bilirubin were both normal by Day 30 post‐transplant. Liver function tests rose again 1 week later (Day 38) and remained elevated for 2 months before normalisation of ALT and bilirubin by Day 96. The biochemical dysfunction during the second and third post‐transplant months was attributed to recurrent hepatitis A virus infection of the transplanted liver. A qualitative HAV PCR test was performed weekly during her inpatient stay and post‐discharge outpatient care and was positive until Day 138. The first negative result was on Day 173 post‐transplant. HAV IgM was still positive when checked on Day 264 post‐transplant. At the time of most recent review (Day 418) the patient was well with normal liver function tests and negative serum HAV PCR.

The results of serum ALT and blood HAV RNA levels are shown in Figure 1. The quantitative HAV measurements were available retrospectively and were not available to aid clinical decisions in real time.

**FIGURE 1:**
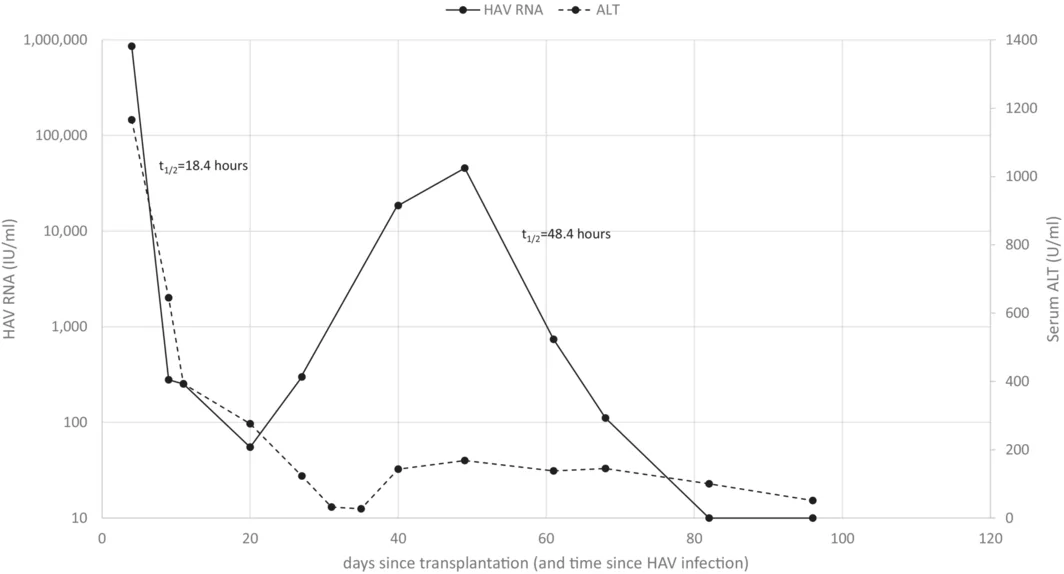
Figure showing the evolution of serum ALT and plasma HAV RNA in a patient who underwent liver transplantation after developing acute liver failure from HAV infection. A classical hepatitis is observed, of duration typically observed in non‐immunosuppressed patients with acute HAV infection. HAV RNA is displayed logarithmically on the *Y*‐axis. HAV RNA = 10 IU/mL represents RNA values beneath the limit of detection (i.e., 6.31 IU/mL).

#### 2.1.2 Quantitative HAV RNA PCR Results

The earliest measurements of blood HAV viral load were made on Days 4 and 9 post‐transplant. RNA levels fell rapidly during that interval with a > 3log_10_ decline observed (virus half‐life 18.4 h). Though the serum ALT fell rapidly during the same period, we believe that the decline of ALT is unrelated to the decline in HAV viral load. Serum ALT is almost inevitably elevated in the early post‐transplant period, mainly reflecting ‘preservation injury’, a term that encompasses the effects of donor circulatory dysfunction and liver preservation after donation of the graft before implantation. This type of liver injury, reflected by high serum ALT, typically resolves very quickly after successful liver transplantation. It seems likely that the decline in HAV RNA in the early post‐transplant period is a consequence of the removal of the HAV‐producing infected liver with implantation of the non‐infected donor liver that provides replication space to act as a sump for circulating HAV. A similar pattern of viral kinetics is observed after liver transplantation for patients with HCV infection. Additional points of observation would more accurately define the HAV kinetics between Days 9 and 20. The effect on HAV production and clearance of events like cellular rejection and augmentation of immunosuppression during that period is uncertain and would require much more intensive sampling. However, beyond Day 20 there is an exponential rise in HAV RNA which peaks about 6 weeks after transplantation. The doubling time for serum HAV during this period was at most 57.2 h, possibly faster. The serum ALT rose to a peak value of 168 IU/mL at the time of the HAV peak then slowly declined to normal range during the period of HAV decline (HAV decline occurred with a half‐life of 48.4 h). The ALT was normal at and after Day 96, at which time the quantitative assay was below the assay's detection threshold.

### 2.2 Case 2. Hepatitis A Infection Acquired in the Early Post‐Transplant Period

#### 2.2.1 Clinical Picture

A 27‐year‐old female was admitted to her local hospital with confusion and vomiting in September 2023. Her blood tests were consistent with acute liver failure secondary to paracetamol poisoning. She was transferred to the Birmingham QEH and registered immediately on the UK National Super‐urgent transplant waiting list. She underwent liver transplantation 3 days after initial presentation. She required perioperative blood transfusion, and a second laparotomy for evacuation of haematoma and to confirm haemostasis was undertaken on post‐transplant Day 11. There was further blood transfusion at the time of the second laparotomy. She then made a good recovery and was discharged from hospital with normal liver function tests on Day 19 post‐transplant. She was reviewed in outpatients on Day 24 (liver function normal) and again on Day 31 (blood tests showing acute liver dysfunction with an ALT of 405 IU/mL). Liver biopsy was performed the following day, and the histology was interpreted as ‘mild acute cellular rejection with prominent apoptosis’. She received additional corticosteroids for 3 days, but without biochemical improvement. On Day 34, we were informed by the NHSBT (National Health Service Blood and Transplant) that one of the blood donors (blood given to our liver recipient at the time of second laparotomy on Day 11 post‐transplant) had developed jaundice and had been diagnosed with acute HAV infection. On Day 35 we confirmed that our transplant patient was HAV viraemic, though serum HAV IgM was negative. In retrospect, the inflammatory infiltrate and the prominent apoptosis seen on liver biopsy 3 days earlier may have principally been attributable to acute HAV infection, thus explaining the lack of biochemical improvement in response to the increased dose of corticosteroids. Subsequently, the patient underwent regular outpatient review and serum remained HAV RNA PCR‐positive until Day 285 post‐transplant, the first negative result being obtained on Day 313 post‐transplant (and with sustained HAV RNA negativity after that date). During her prolonged viraemia we were concerned by the significant second ALT peak and persisting HAV RNA positivity (though only the qualitative measurement was available for prospective decision making). Based on published in vitro data for HAV [16] and supported by the extensive local experience in the use of ribavirin in post‐transplant patients for treatment of HCV and HEV [17, 18], the decision to commence treatment with ribavirin was taken, and the drug commenced on Day 131. A dose of 800 mg/day was given initially, later reduced to 600 mg/day because of anaemia. Biochemical liver function tests improved promptly from that date, possibly suggesting a beneficial effect of treatment. Ribavirin continued until blood and faeces were HAV RNA negative. Blood was HAV IgM negative until Day 61 post‐transplant, positive on Days 61 and 75, and either equivocal or negative on Day 89 and beyond.

The laboratory confirmed that the patient was infected with HAV genotype 1b. The sequence was identical to sequences from the blood donor. The sequence clustered with HAV sequences reported from East Africa.

#### 2.2.2 Quantitative HAV RNA PCR Results

The results of quantitative HAV RNA measurement were available in retrospect and are displayed in Figure 2. Unfortunately, the earliest available serum for quantitative HAV measurement was from Day 31, so the early viral kinetics could not be evaluated. The doubling time measured during the rise in titre between Days 31 and 54 was 55.4 h. If this curve is extrapolated to the early post‐transplant period, then the estimated HAV RNA on Day 11 (the day of transfusion) was 2.7 IU/mL. Beyond Day 54, the HAV viral load was sustained at a level greater than 1 million IU/mL for nearly 2 months before declining rapidly from Day 103 with a calculated half‐life of 99 h (calculated between Days 103 and 131, the day of ribavirin commencement). There was no apparent change in the rate of decay following the introduction of ribavirin. In retrospect, the rapid decline of serum HAV RNA occurred before the commencement of ribavirin and cannot be attributed to ribavirin. The second peak of serum ALT noted on Day 131 post‐transplant occurred during the rapid decline in viral load, consistent with the immunopathogenesis of HAV infection with immune‐mediated clearance of infected hepatocytes.

**FIGURE 2:**
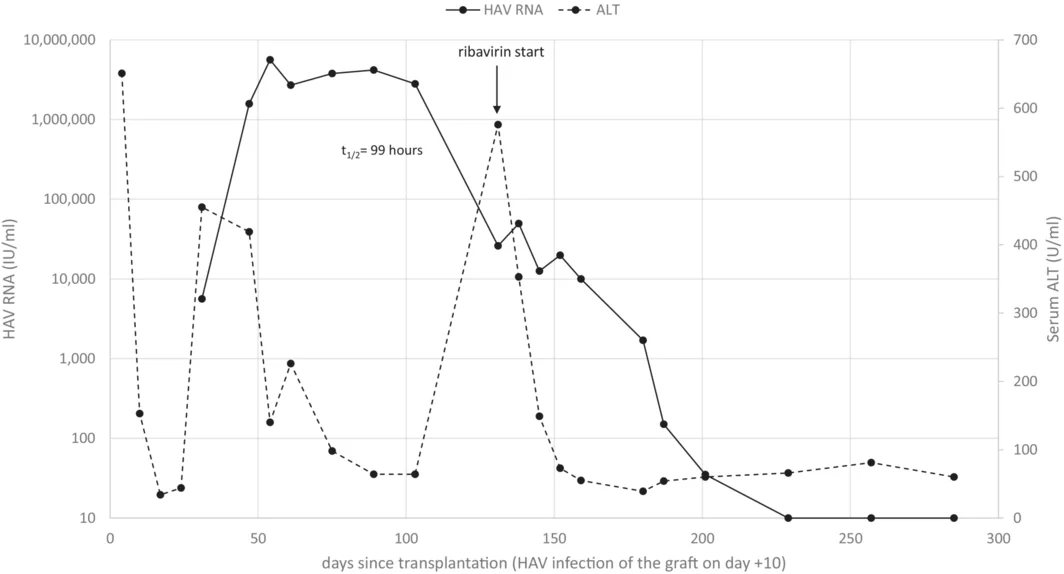
Figure showing the course of serum ALT and plasma HAV RNA in a patient who acquired HAV infection from blood transfusion given on Day 11 after liver transplantation. Compared with the patient represented in Figure 1, the HAV RNA titre rises two logs higher, is maintained at high level for longer, and takes much longer to be cleared from the blood. Though ribavirin was given, the results of HAV RNA measurement suggest that a significant decline in HAV titre began before the drug was given. HAV RNA is displayed logarithmically on the *Y*‐axis. HAV RNA = 10 IU/mL represents RNA values beneath the limit of detection (i.e., 6.31 IU/mL).

## 3 Discussion

Overall, the picture of *recurrent* HAV infection of the transplanted liver (Case 1) is a rapid decline in blood RNA levels in the early post‐transplant period, normalisation of liver function tests during the first month after transplantation, then a rise in HAV RNA which is associated with recurrent liver function disturbance during the second and third post‐transplant months. Using a sensitive qualitative PCR assay, there was no sign of persisting viral infection at 4 months post‐transplant. The pattern of infection observed for the patient with recurrent infection represents infection of the graft by high levels of circulating virus in the setting of an HAV‐specific immune response which was established before transplantation and before the effect of immunosuppression.

By contrast, the picture of *peri‐transplant acquisition* of infection (Case 2) differs from recurrent graft infection (comparison in Figure 3), possibly because of the absence of immunity at the time of graft infection. In comparison with graft reinfection, the timing and rate of rise in serum HAV in acquired infection are almost identical, though the de novo infection emerged slightly later (because it was acquired 10 days post‐transplant). In the absence of immunity, the viral load rose higher and was sustained for much longer than observed for recurrent infection. Also, the eventual duration of detectable HAV RNA was more prolonged in de novo infection.

**FIGURE 3:**
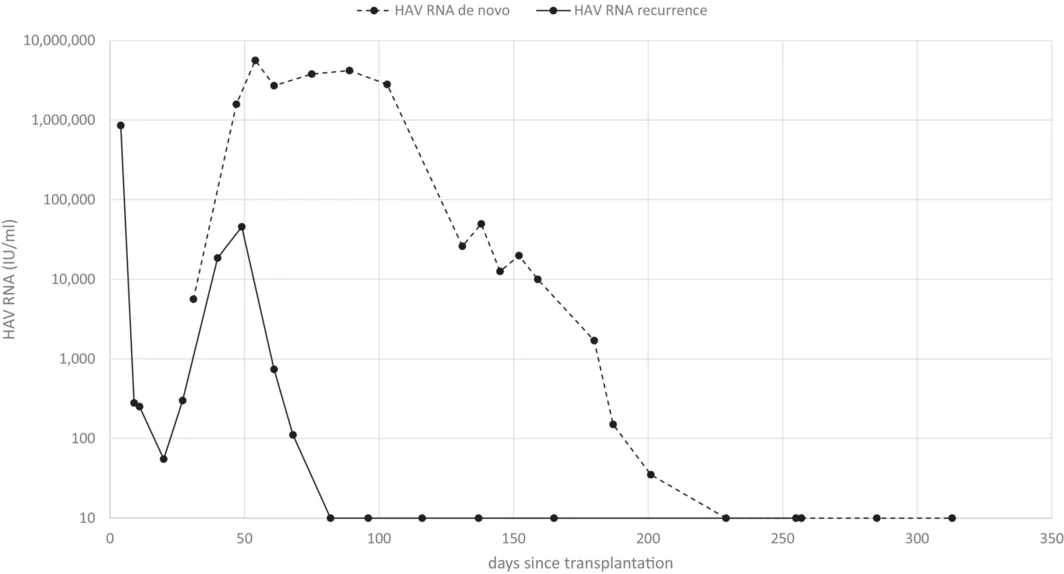
For ease of comparison and contrast, the figure shows the HAV RNA titres of both patients.

We describe 2 liver transplant patients with graft HAV infection. As observed with other types of viral hepatitis, we see a correlation of serum viral RNA levels with clinical events in these patients. According to sequential HAV RNA measurements, the recurrent infection was of much shorter duration than observed for the patient with transfusion‐acquired infection. The duration of HAV viraemia varies in immunocompetent patients and depends on the detection sensitivity of the HAV RNA assay and upon the timing and intensity of the specimen sampling. To summarise the timing and duration of viraemia observed in studies of immunocompetent patients, it seems that sensitive assays will detect serum HAV RNA up to 2 weeks before clinical presentation, and that most patients will become serum HAV RNA negative before 3 months has elapsed after the development of symptoms. In exceptional cases, the HAV RNA can be detected up to 1 year after infection [19, 20, 21, 22, 23, 24]. Our patient with recurrent HAV infection experienced an infection of a duration that is consistent with these published series. The patient with transfusion‐acquired infection suffered a clinical infection of much longer duration (more than 9 months). We speculate that the contrasting durations of infection may be related to an already present and effective immune response in the patient with recurrent infection versus a delayed and probably impaired immune response mounted by the patient who was very immunosuppressed prior to the HAV‐contaminated blood transfusion.

A single study has examined the rate of HAV RNA rise before symptoms [24]. During the initial rapid rise in RNA levels, Schoch and colleagues observed HAV doubling times between 14.1 and 24.7 h. Doubling times for our patients were approximately 60 h, though they may be overestimated because of relatively infrequent sampling during the period of rapid increase in titre. The possible impact of liver preservation injury, cellular rejection and immunosuppression, and of pre‐existing HAV immunity (in Patient 1) on the capacity of the liver to support rapid rates of replication is unknown.

There are few data describing the effect of immunosuppression on the clinical course of HAV infection. Some studies have examined the possible impact of concurrent HIV infection on the clinical pattern of HAV infection. HIV infection may be associated with a longer duration and higher peak of HAV RNA [25, 26], and with a more prolonged serum ALT elevation [27].

A few studies have examined the course of HAV infection in solid organ transplant patients. Eisenbach and colleagues described a case of recurrent HAV infection in a patient transplanted for fulminant HAV infection [4]. The patient developed transaminase elevation on Day 77 post‐transplant and jaundice from Day 118. HCV RNA was detectable in serum from Day 84 until Day 135, though the sensitivity of the assay is not described. A similar duration of infection was observed in our patient who was transplanted for fulminant hepatitis A infection. HAV RNA was detected in her blood until Day 138 post‐transplant.

Foster and colleagues described a case of HAV transmission to a liver/small bowel/pancreas recipient from an HAV viraemic organ donor. The first HAV RNA negative specimens in serum and stool were collected more than 1 year after transplantation [5]. This recipient developed prolonged HAV infection and viraemia despite prior HAV vaccination. This contrasts with the heart and kidney recipients from the same organ donor, also vaccinated pre‐transplant, who showed no sign of HAV acquisition. Jones and colleagues described the outcomes of transplant recipients who received an organ from an HAV‐positive organ donor [28]. The two kidney recipients were still viraemic 6 months after transplantation but achieved sustained HAV RNA negativity during the second half of the first post‐transplant year. Kumar and colleagues describe the clinical outcomes of two kidney recipients from a donor with HAV infection. Serum ALT elevation was observed between Weeks 4 and 8 post‐transplant and remained elevated for an additional 8 to 12 weeks [29]. Werenski and colleagues also described two kidney recipients who acquired hepatitis A from the same donor. For both recipients, serum ALT rose acutely 3 months after transplantation. HAV RNA measurements were not reported [30]. Our second patient, who acquired HAV from perioperative blood transfusion, resembles the pairs of kidney recipients described in these studies. Immunosuppression was first administered at the time of HAV inoculation, often in the absence of pre‐transplant HAV immunity. The pair described by Jones et al. remained viraemic for more than 6 months after transplantation [28].

There is no published human experience with ribavirin in treatment of HAV infection, though an inhibitory effect of the drug has been demonstrated in HAV‐infected foetal Rhesus monkey kidney cells [16]. Our use of ribavirin was in response to prolonged and significant liver dysfunction. At the time of ribavirin treatment, the almost immediate and dramatic normalisation of liver function tests suggested an effective response to the ribavirin. However, it seems likely that the ALT rise preceding ribavirin treatment reflected an emerging and effective immune response to infection with a convincing exponential decline in serum HAV RNA levels occurring at the time of the ALT rise and before initiation of ribavirin. This interpretation was only possible after the event, when the results of sequential quantitative RNA testing were available.

The risk of acquiring HAV from blood transfusion is very low, and NHSBT does not screen donors for evidence of active HAV infection. In the year 2023, our transplanted patient was the only confirmed case of blood donation‐associated HAV transmission in England [31], though this lookback, which requires either donor or recipient to develop symptomatic infection and to notify NHSBT, may underestimate the incidence of transmission. In 2015/2016, a period without major HAV outbreaks in France, the French BTS found that donor HAV positivity was only 5/5.84 million donations, a risk less than 1 per million donors [2].

In summary, we present two patients with HAV infection early post liver transplant, one with recurrent infection and the other with post‐transplant transfusion‐acquired infection. There is a paucity of published literature on the subject. HAV infection is an uncommon indication for liver transplantation. Also, the risk of HAV acquisition from organ donation or blood transfusion is very low. Nucleic acid testing of blood donors for HAV RNA, as now performed in the UK for HBV DNA, HCV RNA and HEV RNA, is feasible. However, the risk of HEV viraemia in blood donors is higher than the risk of HAV viraemia. Also, unlike HEV, HAV infection does not appear to cause chronic infection. Thus, the risk of infection by HAV is very low and long‐term clinical consequences for the recipient are thought to be acceptable. Clearly, it must be perceived that testing of routine blood donations for HAV is not cost‐effective.

## Conflicts of Interest

The authors declare no conflicts of interest.

## Data Availability

The data that support the findings of this study are available on request from the corresponding author. The data are not publicly available due to privacy or ethical restrictions.
